# Supplementary figures and images for: Sp1 mediated the inhibitory effect of glutamate on pulmonary surfactant synthesis
Source: PLoS One. 2023 Aug 9;18(8):e0289530. doi: 10.1371/journal.pone.0289530 (PMC10411742; doi:10.1371/journal.pone.0289530)

Figure 4D

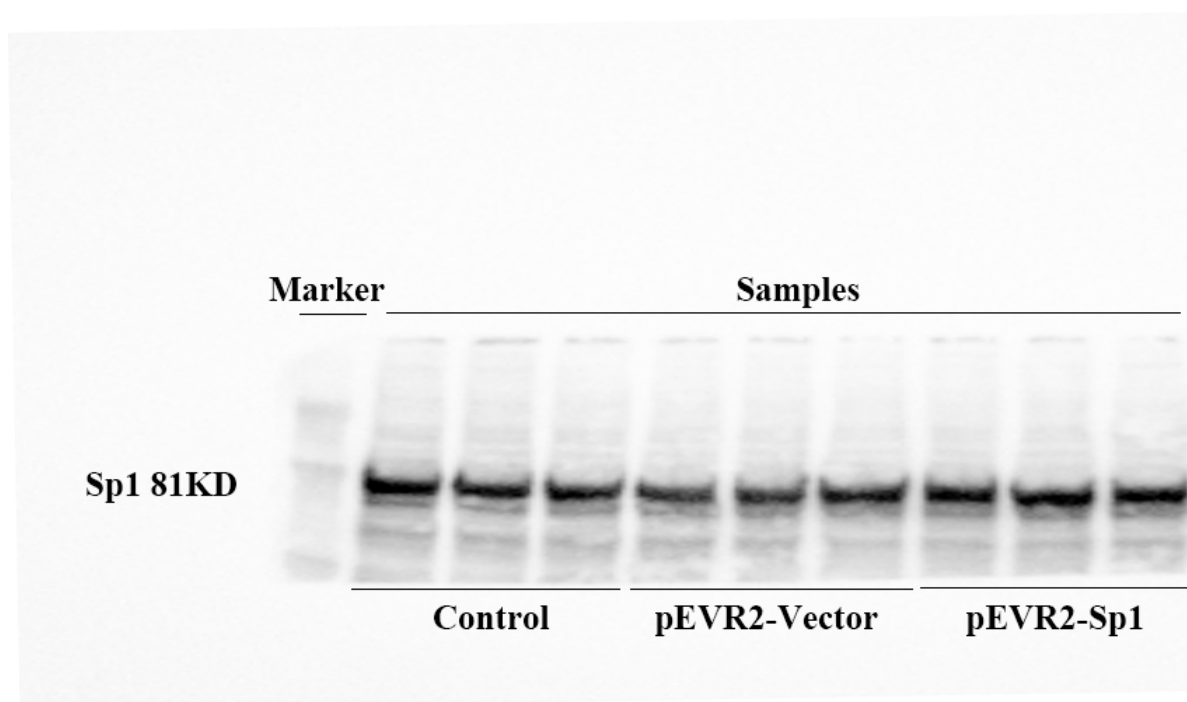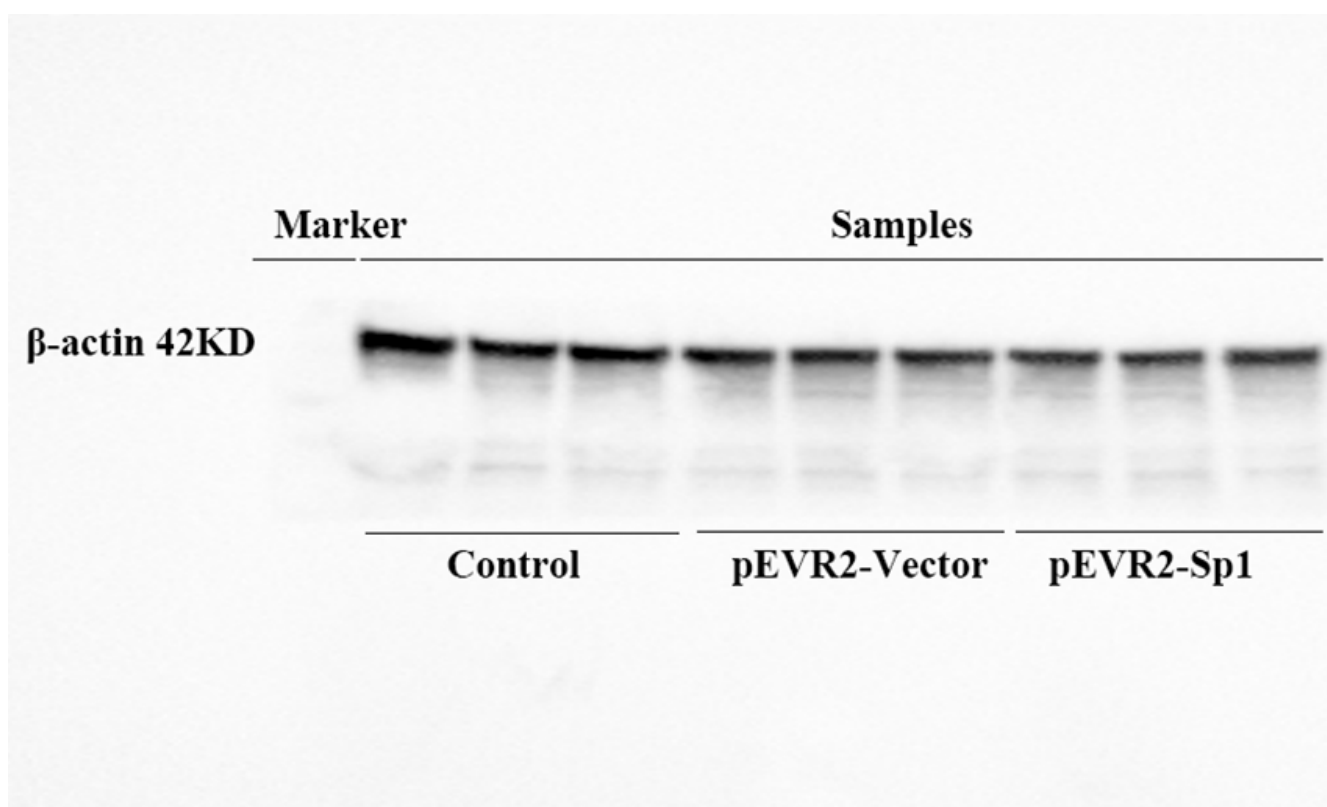

Figure 7A

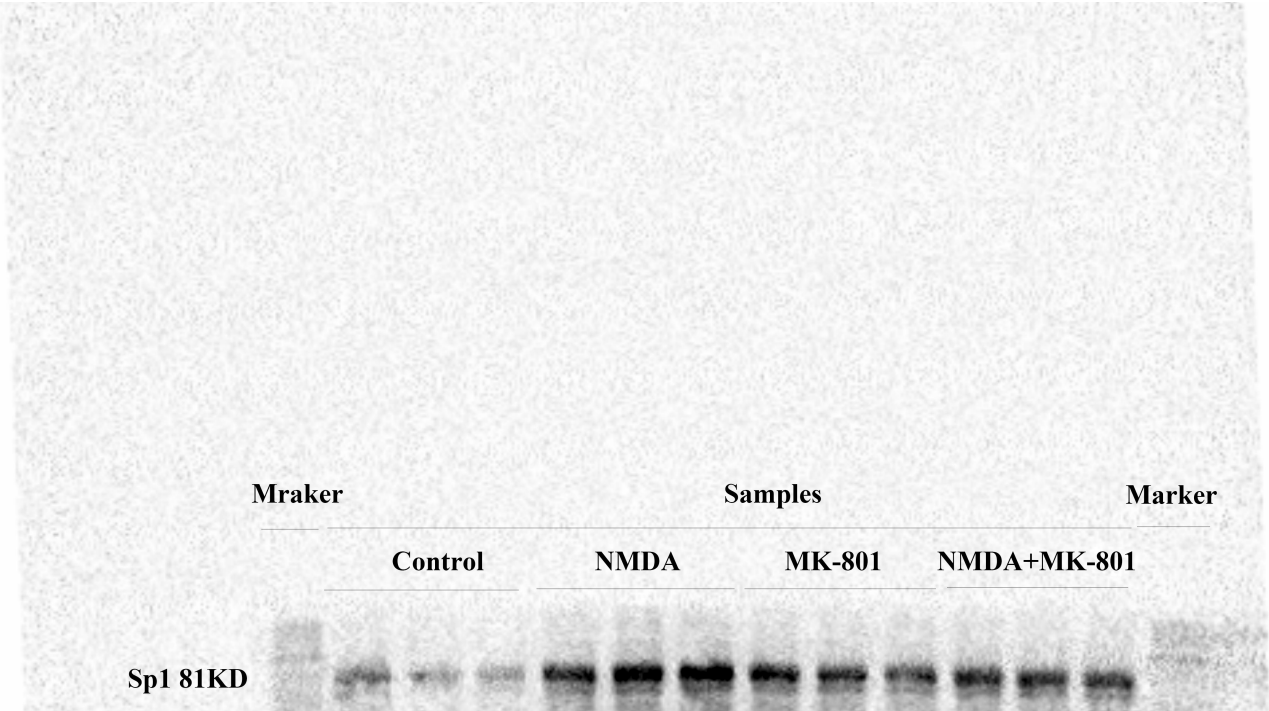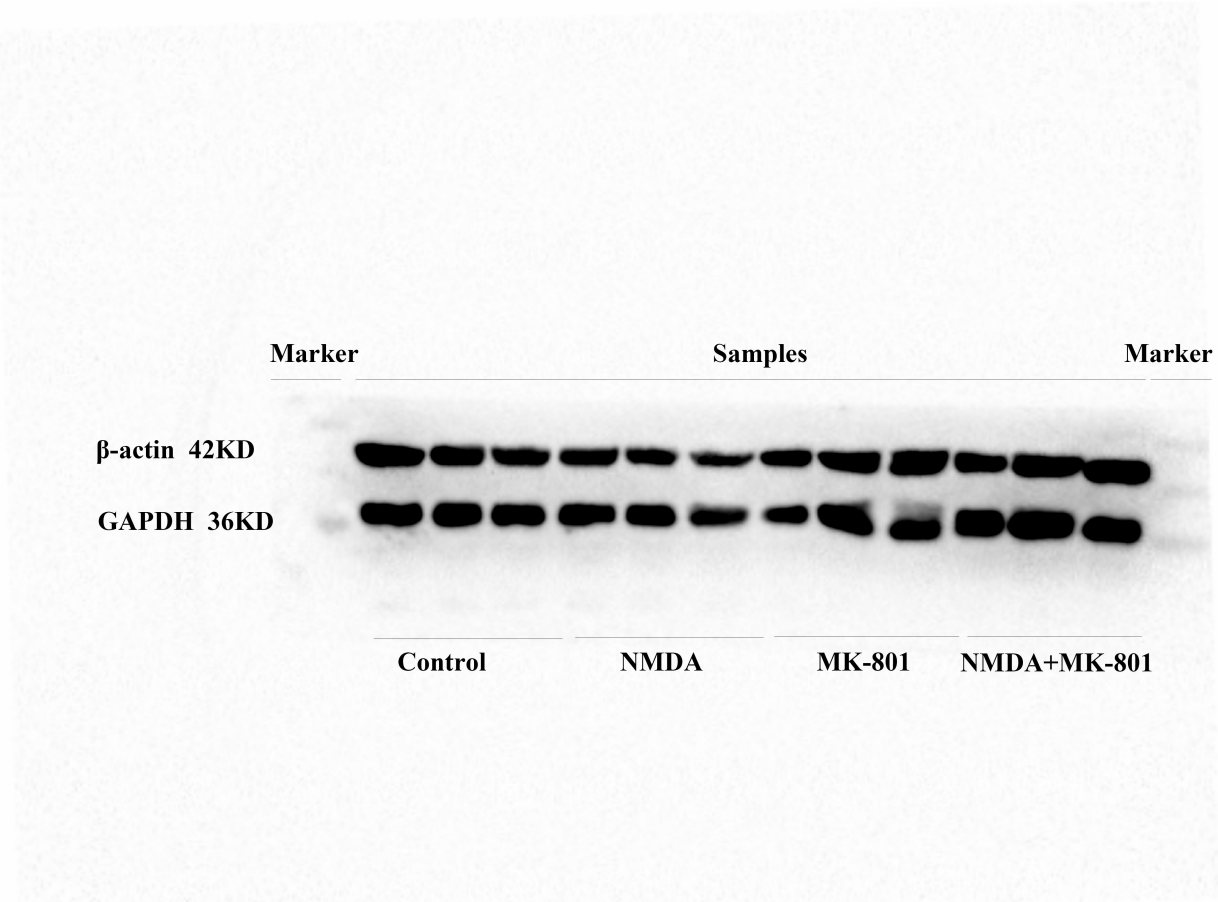

Figure 7C

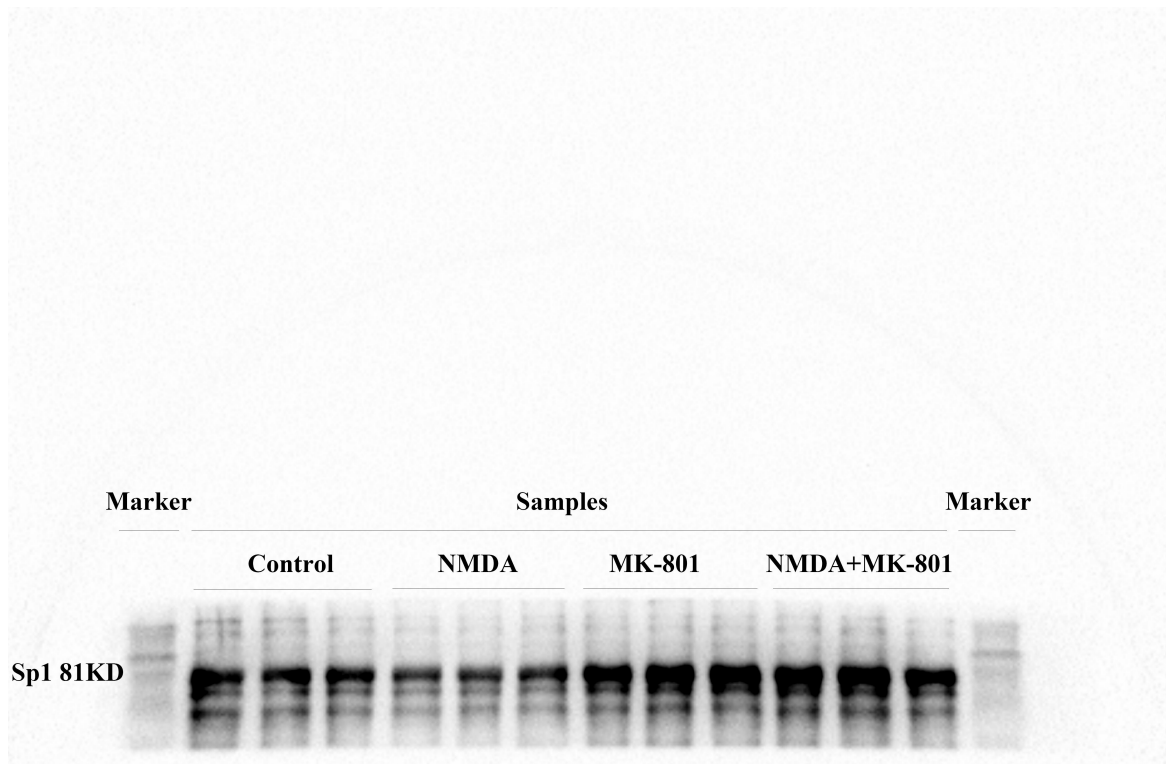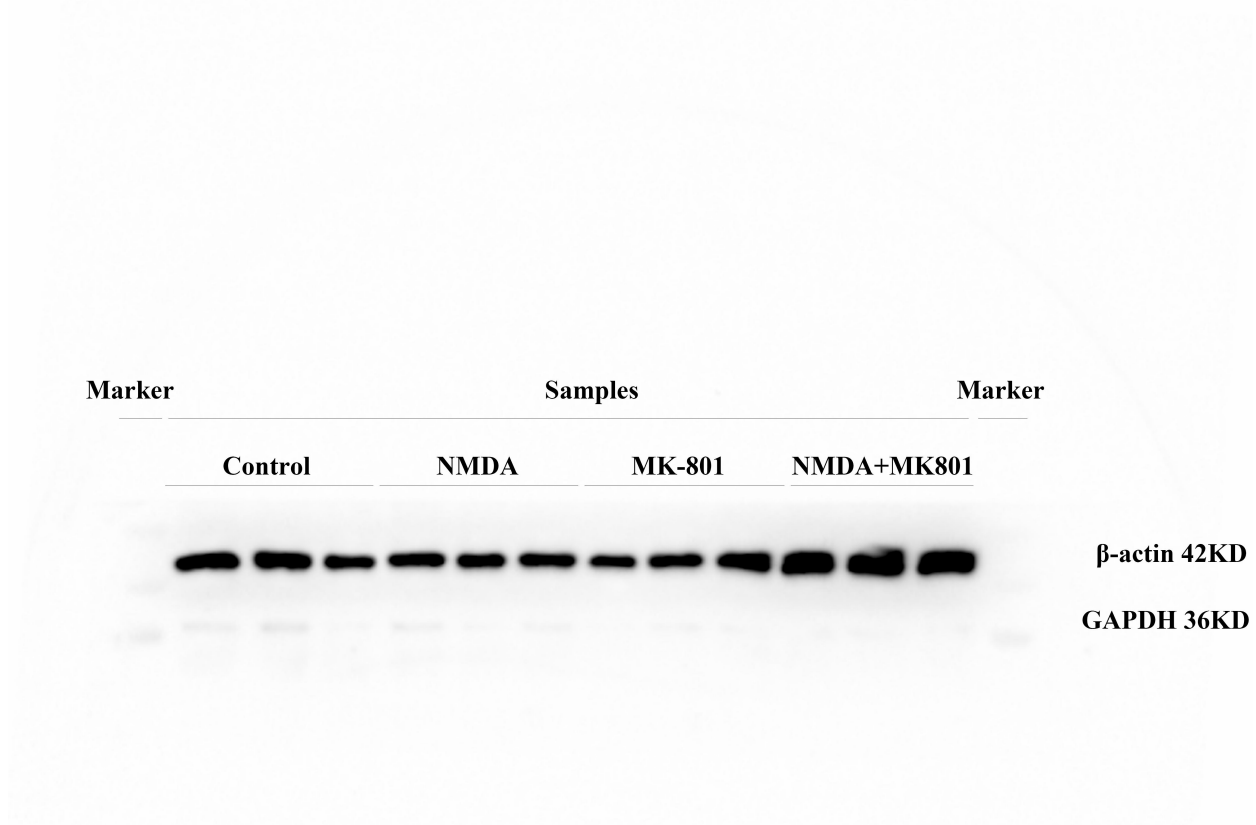

Figure 7C

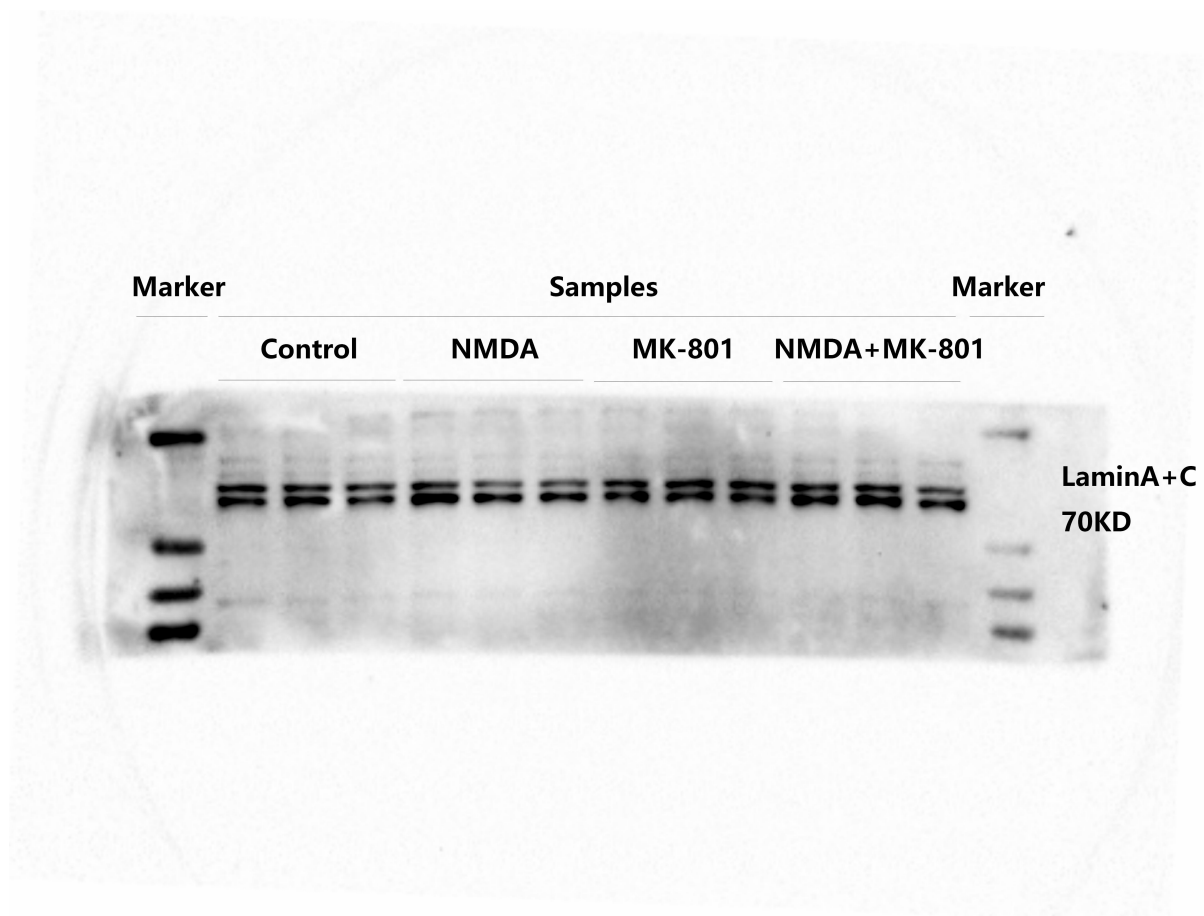

Supplement: S1 Raw images — (PDF) [file pone.0289530.s001.pdf]
