# Supplementary material for: Sp1 mediated the inhibitory effect of glutamate on pulmonary surfactant synthesis
Source: PLoS One. 2023 Aug 9;18(8):e0289530. doi: 10.1371/journal.pone.0289530 (PMC10411742; doi:10.1371/journal.pone.0289530)
Supplement: Attachment — Submitted filename: Response to Reviewers.docx [file pone.0289530.s002.docx]

Title: Sp1 mediated the inhibitory effect of glutamate on pulmonary surfactant synthesis

Authors: Xiao-Hong Li, Jie-Jun Fu, Xiao-Juan Shi, Yun-Na Zhang, Min Shao, Shao-Jie Yue, Chen Li, Zi-Qiang Luo

Manuscript ID: PONE-D-22-33593

Dear Reviewer,

Thank you very much for your letter and valuable comments and suggestions on our manuscript. We have modified and improved our manuscript according to your kind advice and the comments raised by the reviewers, and the amendments are highlighted in red in the revised manuscript. We sincerely hope that the revision is acceptable to be published on PLOS ONE.

Below, please find the comments in black, followed by our responses in red. Exact changes in the manuscript are also presented in red font. The contents in blue font are references.

Thank you very much for all your help and looking forward to hearing from you soon.

With best wishes,

Sincerely yours,

First author: Xiao-Hong Li

Corresponding authors: Zi-Qiang Luo and Chen Li, Email: [luoziqiang@csu.edu.cn](mailto:luoziqiang@csu.edu.cn) and Chen.physiology@outlook.com

Reviewer #1: In this manuscript, Li and colleagues have tried to elucidate the mechanism of phosphatidylcholine biosynthesis under over-activated glutamate/NMDA signaling conditions. Most of what is described in the paper has been previously described in the literature, with the exception possibly being of cytoplasm translocation of Sp1 after NMDA treatment. However, the data from immunofluorescence staining cannot support their wording. Other more readily addressable issues are listed below.

1. (Line 194: Reporter plasmid of CCTα promoter PGL3-CCTα (-1893/+71) …) There is no data presented using this plasmid. Which species (human or rat) of the CCTα promoter sequence is used in reporter assay? Since the DNA sequence between humans and rats is different, the details of Sp1 binding sites within the CCTα promoter should be provided.
2. The data confirming Sp1 overexpression is lacking.
3. (Figure 5-7) The glutamate effects on Sp1 should be examined. Also, rescue experiments should be performed using the NMDAR blocker (MK-801).
4. (Line 285: These results indicated that NMDAR activation inhibited the nuclear translocation of Sp1 …) No data supported this wording unless the protein level of Sp1 is increased or maintained in the cytoplasm fractionation.
5. (Figure 7) The Sp1 immunofluorescence data of NMDA treatment is out of focus. The lens focus is on the mitotic cells. Sp1 is disassociated with mitosis DNA, as described in the previous literature. Also, the quality of image data should be improved and present higher magnification images.
6. Typo: Figure 2B, Glutamae -> Glutamate

Responses: Thank you for your reviews.

1. Reporter plasmid of CCTα core promoter PGL3-CCTα (-169/+71) and pRL-SV40 empty vector with luciferase labeled promoter were from Mallampalli R.K. professor of the university of Iowa. The murine CCTα core promoter fragment (-169/+71) was generated by PCR using the following primers: 5’-TTGTGTGTTTTCACCCCTTATG-3’ (left) and 5’-TCAACTCCTCCAGGCTCCGGT-3’ (right) as described in literature. The core promoter of CCTα gene was localized to a region between -169 and +71 bp, which exhibited strong basal activity comparable with a positive control that contains the simian virus 40 promoter. (Ryan AJ, Fisher K, Thomas CP, Mallampalli RK. Transcriptional repression of the CTP:phosphocholine cytidylyltransferase gene by sphingosine. Biochem J. 2004 Sep 1;382(Pt 2):741-50. doi: 10.1042/BJ20040105. PMID: 15139854; PMCID: PMC1133833.); (Mallampalli RK, Ryan AJ, Carroll JL, Osborne TF, Thomas CP. Lipid deprivation increases surfactant phosphatidylcholine synthesis via a sterol-sensitive regulatory element within the CTP:phosphocholine cytidylyltransferase promoter. Biochem J. 2002 Feb 15;362(Pt 1):81-8. doi: 10.1042/0264-6021:3620081. PMID: 11829742; PMCID: PMC1222362.)

We conducted cell experiments using these plasmids in A549 cells and did obtain such result. We did not consider the issue of species at that time, but it was indeed a very thought-provoking issue. Therefore, we have decided to remove this result from the article to avoid unnecessary misunderstandings, but this does not affect our conclusion. As the reviewer pointed out, the impact of NMDAR activation on Sp1 nuclear translocation is the novelty of our study.

Sp1 is part of a family of approximately 20 proteins that is characterized by having a DNA-binding domain that has 3 Krüppel-like zinc fingers (KLF). Studies have reinforced the central role for Sp1 in activating and promoting the transcription of CCTα gene. (Sugimoto H, Banchio C, Vance DE. Transcriptional regulation of phosphatidylcholine biosynthesis. Prog Lipid Res. 2008 May;47(3):204-20. doi: 10.1016/j.plipres.2008.01.002. Epub 2008 Feb 6. PMID: 18295604.)

Banchio et al. demonstrated that increased binding of the transcription factor Sp1 to the proximal promoter of CCTα is responsible for increased transcription during the S phase. The Sp1 binding element present in position -67/-62 is essential for activation, and the Sp1 site in position -31/-9 is required to enhance transcription. (Banchio C, Schang LM, Vance DE. Activation of CTP:phosphocholine cytidylyltransferase alpha expression during the S phase of the cell cycle is mediated by the transcription factor Sp1. J Biol Chem. 2003 Aug 22;278(34):32457-64. doi: 10.1074/jbc.M304810200. Epub 2003 Jun 6. PMID: 12794070.)

Bakovic et al. also demonstrated that Sp1 binding site 3 (-148/-128) and 1 (-31/-9) might activate or repress transcription of CCTα depends upon the cellular background and Sp1 binding site 2 (-88/-50) would confer a positive regulation independent of the cell context. (Bakovic M, Waite KA, Vance DE. Functional significance of Sp1, Sp2, and Sp3 transcription factors in regulation of the murine CTP:phosphocholine cytidylyltransferase alpha promoter. J Lipid Res. 2000 Apr;41(4):583-94. PMID: 10744779.)

2. We have supplemented the data confirming Sp1 overexpression, as shown in figure 4 (D, E).
3. In the study, we have demonstrated that glutamate down-regulates CCTα expression by specifically activating NMDA receptor, then inhibiting PC synthesis in lung tissues. Next, we further studied the mechanism of CCTα expression downregulation at the cellular level in A549 cells. In cell experiments, we directly treated A549 cells with NMDA, an NMDAR-specific agonist. Glutamate can activate other receptors in addition to NMDA receptor, so instead of treating A549 cells with glutamate, we used the specific agonist NMDA.

The rescue experiments have been performed using the NMDAR blocker (MK-801) (Figure 7).

4. We have added experiments to illustrate this point. the protein levels of Sp1 in the cytoplasm and nucleus of A549 cells after NMDA treatment were detected by Western blot (Figure 7A, B). As shown in figure 7A-D, The expression of Sp1 is increased in the cytoplasmic proteins and decreased in the nuclear proteins after treated with 300 μM NMDA (*P*<0.05, *P*<0.01) (Figure 7C, D). However, 50 μM MK-801 could reverse the increase of Sp1 expression in the cytoplasmic proteins and the decrease in the nuclear proteins induced by NMDA (*P*<0.05, *P*<0.01) (Figure 7C, D).
5. In figure 7E, the immunofluorescence staining of Sp1 after NMDA treatment in A549 cells has been re-detected and the Sp1 immunofluorescence data of NMDA treatment has been re-collected. The quality of image data has been improved. We use a magnification of 20×. Higher magnification images are very blurry.
6. In figure 2B, the “Glutamae” has been modified to “Glutamate”.
