# Supplementary material for: Sp1 mediated the inhibitory effect of glutamate on pulmonary surfactant synthesis
Source: PLoS One. 2023 Aug 9;18(8):e0289530. doi: 10.1371/journal.pone.0289530 (PMC10411742; doi:10.1371/journal.pone.0289530)
Supplement: Attachment — Submitted filename: Response to Reviewers.docx [file pone.0289530.s003.docx]

Title: Sp1 mediated the inhibitory effect of glutamate on pulmonary surfactant synthesis

Authors: Xiao-Hong Li, Jie-Jun Fu, Xiao-Juan Shi, Yun-Na Zhang, Min Shao, Shao-Jie Yue, Chen Li, Zi-Qiang Luo

Manuscript ID: PONE-D-22-33593R1

Dear editor and reviewer,

Thank you very much for your letter and valuable comments and suggestions for our manuscript. We have modified and improved our manuscript according to your kind advice and the comments raised by the reviewers, and the amendments are highlighted in red in the revised manuscript. We sincerely hope that the revision is acceptable to be published on PLOS ONE.

Below, please find the comments in black, followed by our responses in red. Exact changes in the manuscript are also presented in red font.

Thank you very much for all your help and looking forward to hearing from you soon.

With best wishes,

Sincerely yours,

First author: Xiao-Hong Li

Corresponding authors: Zi-Qiang Luo and Chen Li, Email: [luoziqiang@csu.edu.cn](mailto:luoziqiang@csu.edu.cn) and Chen.physiology@outlook.com

Reviewer #1: Although the authors have responded to most of my previous comments and revised the manuscript accordingly, the data quality issues remain.

Responses: Thank you for your reviews.
1. In Figures 4D and 4E, the Western blotting data can not correspond to quantified data.

Response: Although the trend of band changes is not obvious when viewed with the naked eye, after statistical analysis, there are statistical differences between different groups. The analysis data of the bar chart is shown in the tables below (Table1-3). The analysis software we used is Bio Rad's Image Lab software. The bar chart and statistical analysis were performed using Image-Pro Plus 6.0 software.

| Table 1. Analysis of the grayscale values of Sp1 bands in figure 4D. | | | | | | | | | |
| --- | --- | --- | --- | --- | --- | --- | --- | --- | --- |
| No. | Volume (Int) | Adjust volume (Int) | Average background (Int) | Pixels | Min (Int) | Max (Int) | Mean (Int) | standard deviation | Region (mm^2^) |
| 1 | 8,224,181.00 | 4,997,169.59 | 13,064.82 | 247 | 5,559.00 | 65,534.00 | 33,296.28 | 15,434.72 | 18.407109 |
| 2 | 6,827,509.00 | 4,234,016.26 | 10,499.97 | 247 | 4,569.00 | 55,962.00 | 27,641.74 | 14,738.05 | 18.407109 |
| 3 | 6,752,659.00 | 4,006,309.59 | 11,118.82 | 247 | 5,583.00 | 57,328.00 | 27,338.70 | 14,783.47 | 18.407109 |
| 4 | 6,367,799.00 | 3,597,007.49 | 11,217.78 | 247 | 6,738.00 | 48,469.00 | 25,780.56 | 11,662.64 | 18.407109 |
| 5 | 6,139,306.00 | 3,229,446.22 | 11,780.81 | 247 | 3,300.00 | 49,925.00 | 24,855.49 | 12,861.86 | 18.407109 |
| 6 | 7,519,719.00 | 3,659,123.53 | 15,629.94 | 247 | 7,529.00 | 55,692.00 | 30,444.21 | 13,733.55 | 18.407109 |
| 7 | 7,183,335.00 | 3,939,229.74 | 13,134.03 | 247 | 5,347.00 | 58,069.00 | 29,082.33 | 15,529.68 | 18.407109 |
| 8 | 7,836,128.00 | 4,349,821.07 | 14,114.60 | 247 | 8,003.00 | 58,379.00 | 31,725.21 | 15,848.29 | 18.407109 |
| 9 | 7,495,900.00 | 4,205,889.06 | 13,319.88 | 247 | 6,686.00 | 60,208.00 | 30,347.77 | 15,766.36 | 18.407109 |

| Table 2. Analysis of the grayscale values of β-actin bands in figure 4D. | | | | | | | | | |
| --- | --- | --- | --- | --- | --- | --- | --- | --- | --- |
| No. | Volume (Int) | Adjust volume (Int) | Average background (Int) | Pixels | Min (Int) | Max (Int) | Mean (Int) | standard deviation | Region (mm^2^) |
| 1 | 7,160,003.00 | 5,203,131.24 | 8,153.63 | 240 | 2,517.00 | 65,534.00 | 29,833.35 | 17,385.53 | 17.88545 |
| 2 | 5,265,437.00 | 3,114,860.53 | 8,960.74 | 240 | 2,427.00 | 54,840.00 | 21,939.32 | 15,734.69 | 17.88545 |
| 3 | 5,683,049.00 | 3,709,197.24 | 8,224.38 | 240 | 2,019.00 | 60,902.00 | 23,679.37 | 17,622.91 | 17.88545 |
| 4 | 5,000,967.00 | 2,996,801.12 | 8,350.69 | 240 | 1,664.00 | 53,427.00 | 20,837.36 | 15,018.29 | 17.88545 |
| 5 | 5,062,446.00 | 3,262,615.41 | 7,499.29 | 240 | 2,173.00 | 45,945.00 | 21,093.53 | 13,128.59 | 17.88545 |
| 6 | 4,895,367.00 | 3,134,342.29 | 7,337.60 | 240 | 2,077.00 | 45,375.00 | 20,397.36 | 12,668.68 | 17.88545 |
| 7 | 4,468,360.00 | 2,934,925.88 | 6,389.31 | 240 | 1,882.00 | 42,366.00 | 18,618.17 | 12,509.06 | 17.88545 |
| 8 | 4,084,113.00 | 2,595,121.24 | 6,204.13 | 240 | 1,812.00 | 42,545.00 | 17,017.14 | 12,356.63 | 17.88545 |
| 9 | 4,867,808.00 | 3,327,117.41 | 6,419.54 | 240 | 1,789.00 | 42,307.00 | 20,282.53 | 12,160.04 | 17.88545 |

| Table 3. Statistical data of the bar chart in figure 4E. | | | | |
| --- | --- | --- | --- | --- |
| Groups | No. | Sp1 mean (Int) | β-actin mean (Int) | Sp1/β-actin |
| Control | 1 | 33,296.28 | 29,833.35 | 1.116075935 |
|  | 2 | 27,641.74 | 21,939.32 | 1.259917618 |
|  | 3 | 27,338.70 | 23,679.37 | 1.154536605 |
| pEVR2-Vector | 4 | 25,780.56 | 20,837.36 | 1.237227732 |
|  | 5 | 24,855.49 | 21,093.53 | 1.178346904 |
|  | 6 | 30,444.21 | 20,397.36 | 1.492556034 |
| pEVR2-Sp1 | 7 | 29,082.33 | 18,618.17 | 1.562040369 |
|  | 8 | 31,725.21 | 17,017.14 | 1.864309704 |
|  | 9 | 30,347.77 | 20,282.53 | 1.496251616 |

2. In Figures 7B and 7D, which internal control is used for quantitative analysis? Although β-actin is also present in the nucleus, it is not suitable to be used as a control for nuclear protein samples.

Response: We have used the rabbit monoclonal antibody to Lamin A + Lamin C (ab108595), which is the nuclear envelope marker. The strip diagram of LaminA+C is shown in the figure 7C. In figures 7B, the GAPDH is used for quantitative analysis in the cytoplasmic proteins. In figures 7D, the Lamin A+C is used for quantitative analysis in the nuclear proteins. They are reflected in the annotation of the vertical coordinates in Figures 7B and 7D.
3. In Figure 7E, the image data is highly blurry, which can not support their statement of Sp1 translocation.

Response: In figure 7E, the image data we uploaded to the submission system is clear. The image data in the PDF of the manuscript is relatively blurry, possibly due to reduced pixels. We suggest that you download the original images in the review system to view it, which may be better. In figure 7E, the green color is Sp1, while the blue color is the nucleus. The green color is Sp1, while the blue color is the nucleus. Compared to the control group, the NMDA treatment group showed a decrease in overlapping green and blue cells, while MK-801 could alleviate the reduction caused by NMDA treatment. This indicates a decrease in Sp1 in the nucleus after NMDA treatment.
