# Supplementary material for: Sp1 mediated the inhibitory effect of glutamate on pulmonary surfactant synthesis
Source: PLoS One. 2023 Aug 9;18(8):e0289530. doi: 10.1371/journal.pone.0289530 (PMC10411742; doi:10.1371/journal.pone.0289530)
Supplement: Attachment — Submitted filename: Response to Reviewers.docx [file pone.0289530.s004.docx]

Title: Sp1 mediated the inhibitory effect of glutamate on pulmonary surfactant synthesis

Authors: Xiao-Hong Li, Jie-Jun Fu, Xiao-Juan Shi, Yun-Na Zhang, Min Shao, Shao-Jie Yue, Chen Li, Zi-Qiang Luo

Manuscript ID: PONE-D-22-33593R2

Dear editor and reviewers,

Thank you very much for your letter and valuable comments and suggestions for our manuscript. We have modified and improved our manuscript according to your kind advice and the comments raised by the reviewers. Below, please find the comments in black, followed by our responses in red point by point. Exact changes in the 'Revised Manuscript with Track Changes' file are also presented in red font. We sincerely hope that the revision is acceptable to be published on PLOS ONE. Thank you very much for all your help and looking forward to hearing from you soon.

Reviewer #1: The authors claim that the NMDA treatment group showed decreased colocalization of Sp1 and nucleus, while MK-801 could alleviate the reduction caused by NMDA treatment. But they choose inappropriate data that may lead readers to confusion and misunderstanding. Because one cell expresses a highly green fluorescence signal, the field intensities lead to saturation in a few milliseconds, resulting in the other cells exhibiting a lower fluorescence signal. The author might explain that the expression of Sp1 was decreased under NMDA treatment, but this figure aims to illustrate the finding of nuclear-cytosol translocation of Sp1. They should provide three images representing blue (DAPI), green (Sp1), or yellow (merge) fluorescent channels, just like in the first version of this article, and quantify the percentage of cells with nuclear and cytoplasmic Sp1. Although the result of nuclear and cytoplasmic protein extraction could support their finding (Effects of NMDA on Sp1 nuclear translocation in A549 cells), the immunofluorescence staining data are not.

Author response #1: Thank you very much for the valuable feedback from the reviewer. The opinions of the reviewer are very important and reasonable. The Western blot results of nuclear and cytoplasmic protein extraction can totally support our finding that NMDA inhibits Sp1 nuclear translocation in A549 cells. The results of immunofluorescence staining are not very suitable. In order to avoid misleading the readers, we have decided not to present this result in the article. This does not affect our research conclusion (Effects of NMDA on Sp1 nuclear translocation in A549 cells). We have removed the contents about Sp1 fluorescence staining in the article.

With best wishes,

Sincerely yours,

First author: Xiao-Hong Li

Corresponding authors: Zi-Qiang Luo and Chen Li,

Email: [luoziqiang@csu.edu.cn](mailto:luoziqiang@csu.edu.cn) and Chen.physiology@outlook.com
